# ^99m^Tc-Sestamibi/^123^I Subtraction SPECT/CT in Parathyroid Scintigraphy: Is Additional Pinhole Imaging Useful?

**DOI:** 10.1155/2017/2712018

**Published:** 2017-10-18

**Authors:** Virpi Tunninen, Pekka Varjo, Tomi Kauppinen, Anu Holm, Hannu Eskola, Marko Seppänen

**Affiliations:** ^1^Department of Nuclear Medicine, Satakunta Central Hospital, Sairaalantie 3, 28500 Pori, Finland; ^2^Helsinki University Hospital, HUS Medical Imaging Center, P.O. Box 340, 00029 Helsinki, Finland; ^3^Faculty of Biomedical Sciences, Tampere University of Technology, P.O. Box 692, 33101 Tampere, Finland; ^4^Regional Imaging Center, Tampere University Hospital, P.O. Box 2000, 33521 Tampere, Finland; ^5^Turku PET Centre, Turku University Hospital, P.O. Box 52, 20521 Turku, Finland; ^6^Department of Clinical Physiology and Nuclear Medicine, Turku University Hospital, P.O. Box 52, 20521 Turku, Finland

## Abstract

**Objectives:**

This retrospective study evaluated whether the use of additional anterior ^99m^Tc-sestamibi/^123^I pinhole imaging improves the outcome of ^99m^Tc-sestamibi/^123^I subtraction SPECT/CT in parathyroid scintigraphy (PS).

**Materials and Methods:**

PS using simultaneous dual-isotope subtraction methods and an acquisition protocol combining SPECT/CT and planar pinhole imaging was performed for 175 patients with primary or secondary hyperparathyroidism. All patients who proceeded to surgery with complete postsurgery laboratory findings were included in this study (*n* = 94). SPECT/CT images alone and combined with pinhole images were evaluated.

**Results:**

There were 111 enlarged parathyroid glands of which 104 and 108 glands were correctly visualized by SPECT/CT (seven false positives) or SPECT/CT with pinhole (three false positives), respectively. Both sensitivity and specificity were higher with combined SPECT/CT with pinhole than with SPECT/CT alone (97% versus 94% and 99% versus 98%, resp., not significant). The false-positive rate was 6% with SPECT/CT and decreased to 3% using combined SPECT/CT with pinhole.

**Conclusion:**

^99m^Tc-sestamibi/^123^I subtraction SPECT/CT is a highly sensitive and specific protocol for PS. The use of additional anterior pinhole imaging increases both sensitivity and specificity of PS, although this increase is not statistically significant.

## 1. Introduction

Primary hyperparathyroidism is a common endocrine disorder caused by one or more hyperfunctioning parathyroid glands. Hyperfunctioning parathyroid glands secrete excess amounts of parathyroid hormone, which raises the blood calcium level. Secondary hyperparathyroidism, which is most commonly seen in patients with chronic kidney failure, refers to parathyroid gland hyperfunction in response to low blood calcium levels. Patient symptoms and laboratory findings contribute to identifying candidates for surgery, which is the only curative treatment for these indications [1].

Preoperative localization of hyperfunctioning parathyroid glands is highly recommended to define surgical strategy, select patients for minimally invasive surgery, and reduce the rate of surgical conversion or failure. Imaging protocols should effectively provide morphologic and functional information in a noninvasive and cost effective manner [2].

Today, scintigraphic imaging is usually performed using the dual-phase method, where only ^99m^Tc-sestamibi is used with early and late phase acquisitions. This method is based on the assumption that the kinetics of ^99m^Tc-sestamibi is different in thyroid and parathyroid glands, thereby revealing hyperfunctioning parathyroid glands in late phase images. This dual-phase method is attractive for its technical simplicity and low cost [3]. Unfortunately, this technique has limits for detecting parathyroid adenomas with rapid ^99m^Tc-sestamibi clearance and low sensitivity for localizing multiple parathyroid glands due to parathyroid hyperplasia or differentiating thyroid nodules from parathyroid lesions [4].

These pitfalls can be avoided with more technically demanding dual-isotope subtraction methods with ^123^I and ^99m^Tc-sestamibi. Thyroid-seeking ^123^I is acquired simultaneously with ^99m^Tc-sestamibi, and normalized ^123^I images are subtracted from ^99m^Tc-sestamibi images. Residual activity in subtraction images reveals hyperfunctioning parathyroid glands. The advantages of this method have been shown in several recent studies, and it is also recommended in the European Association of Nuclear Medicine (EANM) guidelines [5–10].

Several acquisition techniques can be used with each method. Both planar imaging (with parallel hole or pinhole collimators) and three-dimensional (3D) imaging (SPECT and SPECT/CT) are widely used. Although the trend is towards using 3D acquisition techniques, pinhole imaging remains a recommended part of any parathyroid imaging protocol and it is still considered superior owing to its much higher spatial resolution [9–14].

However, pinhole imaging lacks the 3D anatomical information offered by modern SPECT/CT imaging which may be very helpful in planning the surgical approach and even in shortening surgical times [4, 14, 15]. These techniques are often combined in clinical practice to increase sensitivity [3, 12, 16, 17].

It would be tempting to simplify the parathyroid imaging protocol using only SPECT/CT imaging, but this would have to be performed without compromising sensitivity or specificity. Some preliminary results have already been published using the dual-isotope method where SPECT/CT results were equivalent or even better than pinhole [7, 11, 18]. However, contrasting data showing pinhole imaging alone yielded better results than SPECT/CT alone or even combined with pinhole have been reported [13].

The aim of this study was to compare two protocols for parathyroid scintigraphy in the same patients with primary or secondary hyperparathyroidism: ^99m^Tc-sestamibi/^123^I subtraction SPECT/CT alone or combined with additional pinhole imaging.

## 2. Materials and Methods

### 2.1. Patients

From March 2011 through June 2016, 175 patients were referred for parathyroid scintigraphy. All patients had biochemical evidence of hyperparathyroidism (elevated total serum calcium (Ca-ion) and parathyroid hormone level [PTH]). From this original cohort, patient data were included in this retrospective study if the patient proceeded to surgery and if postoperative Ca-ion and PTH results were available. Surgical and histopathological findings with postoperative Ca-ion and PTH levels were used as the gold standard.

The final study group consisted of 94 patients (27 men and 67 women) with a mean age of 64.0 years (range, 22.3–83.6 years). Of these, 84 patients had primary hyperparathyroidism (pHPT), and 10 had secondary hyperparathyroidism (sHPT). This study was exempt from approval by the institutional review board. The requirement for informed consent was waived.

### 2.2. Parathyroid Scintigraphy Protocols

Patients received 20 MBq of ^123^I intravenously. Two hours later, 750 MBq of ^99m^Tc-sestamibi was injected. SPECT/CT acquisition was started 10 minutes after the ^99m^Tc-sestamibi administration. All SPECT/CT acquisitions were performed with Siemens Symbia T or Siemens Intevo T2 imaging systems (Siemens, Erlangen, Germany). Immediately after the SPECT/CT acquisition, a static pinhole image of the neck was acquired with a SKYLight gamma camera (Philips Healthcare, USA) or with Siemens Symbia or Intevo T2 systems (Siemens, Erlangen, Germany) beginning in 2015. All acquisition parameters are shown in Table 1.

### 2.3. Surgery and Histologic Analysis

An endocrine surgeon performed all operations and was aware of all initial scintigraphic results prior to surgery. The mean interval between scintigraphy and surgery was 164 days (range, 8–1065 days). Histopathological analysis was performed on all excised tissue.

### 2.4. Image Processing

^99m^Tc-sestamibi and ^123^I SPECT images (with attenuation correction) were anonymized and reconstructed on the Siemens Syngo workstation using the FLASH 3D-algorithm (eight iterations, eight subsets, Gaussian 9.00 filter). ^123^I SPECT images were multiplied by a normalization factor (defined as the ratio of the corresponding thyroid maximum voxel counts in ^99m^Tc-sestamibi and ^123^I SPECT images with manual adjustment based on visual evaluation, if necessary) to create normalized ^123^I SPECT images that were then subtracted from ^99m^Tc-sestamibi SPECT images. The final image sets consisted of ^99m^Tc-sestamibi SPECT images, ^123^I SPECT images, and subtraction SPECT images. Images were displayed in three axes (transverse, coronal, and sagittal) alone, fused together (as ^123^I fused with subtraction images), or fused with CT images. Static pinhole images were anonymized and processed in a Siemens Syngo workstation. Subtraction images were created in a similar manner as previously described. The final image set consisted of ^99m^Tc-sestamibi images, ^123^I images, and subtraction images. All image processing was performed by an experienced medical physicist.

### 2.5. Image Interpretation

Anonymized imaging data were reviewed for the purpose of this study by an experienced nuclear medicine physician who had no previous knowledge of images, patient information, or surgery results. The review was performed in two phases: the SPECT/CT data were reviewed first and each quadrant in relation to the thyroid gland (right upper, right lower, left upper, left lower, other/ectopic) was classified on a two-point scale (negative or positive). The image review criteria for positive finding were as follows: clear abnormal residual activity on the subtraction SPECT images that had an anatomic correlation in the CT images. Thereafter, the pinhole images were added next to the SPECT/CT data and images were reviewed together again in similar manner.

To estimate the sensitivity, specificity, and accuracy for the localization for each image set, findings were classified as true positive, false positive, true negative, or false negative with histologic analysis and postoperative laboratory findings as the reference standard. For each patient, five scores, one for each quadrant plus other (referring as ectopic), were assigned. The false-positive image rate was defined as the ratio of false positives to the sum of true positives plus false positives.

### 2.6. Statistical Analysis

The sensitivity, specificity, and accuracy of each image set were calculated. McNemar test was performed to compare the sensitivities, specificities, and accuracies between the two image sets. A *p* value less than 0.05 was considered statistically significant. Statistical analyses were conducted using SPSS v.21 (IBM Corp., Armonk, NY).

## 3. Results

### 3.1. Surgical and Histological Findings

Altogether, 111 enlarged glands were found in 94 patients in surgery. Seventy-nine patients had a solitary parathyroid adenoma, one had parathyroid carcinoma, 12 had double adenomas, and two had multiglandular disease (one patient with three enlarged glands and one patient with four enlarged glands). The mean weight of the abnormal parthyroid glands was 1.42 g (0.07–11 g). The smallest visible gland was 0.07 g in both image sets.

### 3.2. Imaging Results

**Figure 1:**
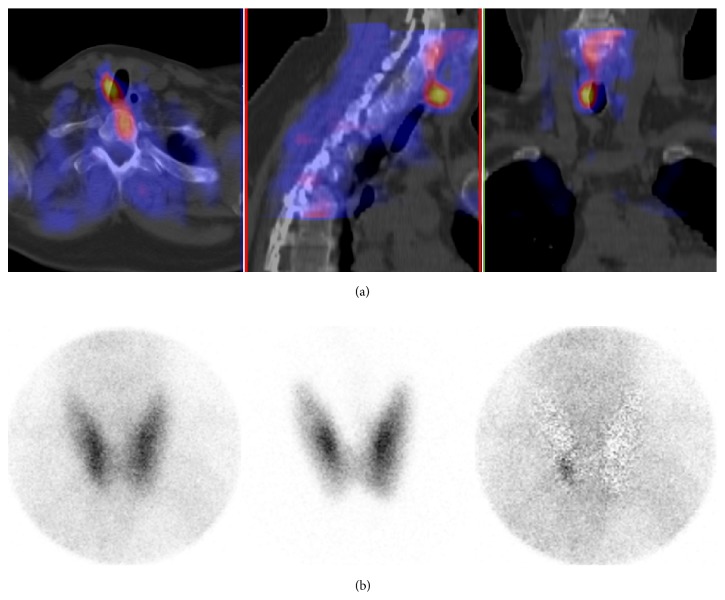
An example of a true-positive finding with SPECT/CT and with pinhole in primary hyperparathyroidism. (a) Fusion SPECT/CT images. (b) Anterior pinhole images (*left*: ^99m^Tc-sestamibi image;* middle*: ^123^I image;* right*: subtraction image). A 230 mg adenoma (arrows) was resected behind the lower right quadrant of the thyroid.

^99m^Tc-sestamibi/^123^I subtraction SPECT/CT was able to locate 104 enlarged glands correctly, and missed seven enlarged glands that were located in surgery. Combined ^99m^Tc-sestamibi/^123^I subtraction SPECT/CT with additional pinhole imaging was able to locate 108 enlarged glands and missed three enlarged glands. A typical patient case with a true-positive finding is shown in Figure 1.

The sensitivity and specificity were slightly higher with SPECT/CT and pinhole combined than with SPECT/CT alone, but the difference was not significant. The false-positive rate was 6% with SPECT/CT and decreased to 3% with combined SPECT/CT and pinhole. Sensitivity and specificity were also calculated separately for patients with sHPT or pHPT. As expected, sensitivity was lower for patients with sHPT compared to patients with pHPT. There were no false-positive findings in patients with sHPT. Results are depicted in Table 2.

### 3.3. False-Negative Findings

Four parathyroid glands were faintly visible in SPECT/CT alone but were classified falsely as negative with SPECT/CT alone. With additional pinhole imaging, these were correctly classified as true positive

Three parathyroid glands were falsely classified as negative in SPECT/CT and were also not visible in pinhole images. Two of these were small hyperplastic glands according to histologic report, and one was a small atypical adenoma. These three patients had more than one hyperplastic gland or adenoma, and smallest glands were not visualized in these cases. Patient cases with false-negative findings are presented in Table 3.

### 3.4. False-Positive Findings

Four parathyroid glands were classified falsely as positive with SPECT/CT alone but were correctly classified as true negative with additional pinhole imaging. Two of these were owing to uneven iodine uptake, with iodine-negative thyroid nodules more clearly visible in pinhole images. Two false-positive findings were subtraction artifacts, not visible in pinhole images.

Three false-positive findings in patients with complex multiglandular disease that were classified with SPECT/CT alone remained falsely classified with the additional pinhole imaging.

First patient (case number 5a/5b in Table 3) had a true-positive adenoma at the upper left quadrant that was attached to a large thyroid cyst. There was weak residual activity at the lower left quadrant on both SPECT/CT and pinhole imaging classified as positive, but no adenoma was found during surgery (normal parathyroid gland was seen). There was also a small adenoma at upper right quadrant, but it was not visible in either SPECT/CT or pinhole imaging and was classified as a false negative. Postoperative Ca-ion and PTH were normalized for this patient.

**Figure 2:**
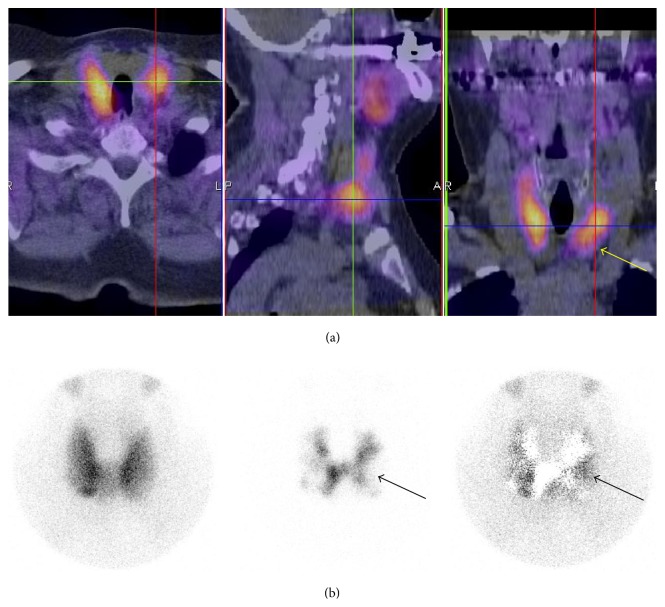
An example of a false-positive finding (case number seven in Table 3) on a ^99m^Tc-sestamibi/^123^I SPECT/CT due to an uneven iodine uptake and iodine-negative nodule on a left thyroid gland (arrows). (a) Fusion SPECT/CT images. (b) Anterior pinhole images (*left*: ^99m^Tc-sestamibi image;* middle*: ^123^I image;* right*: subtraction image).

**Figure 3:**
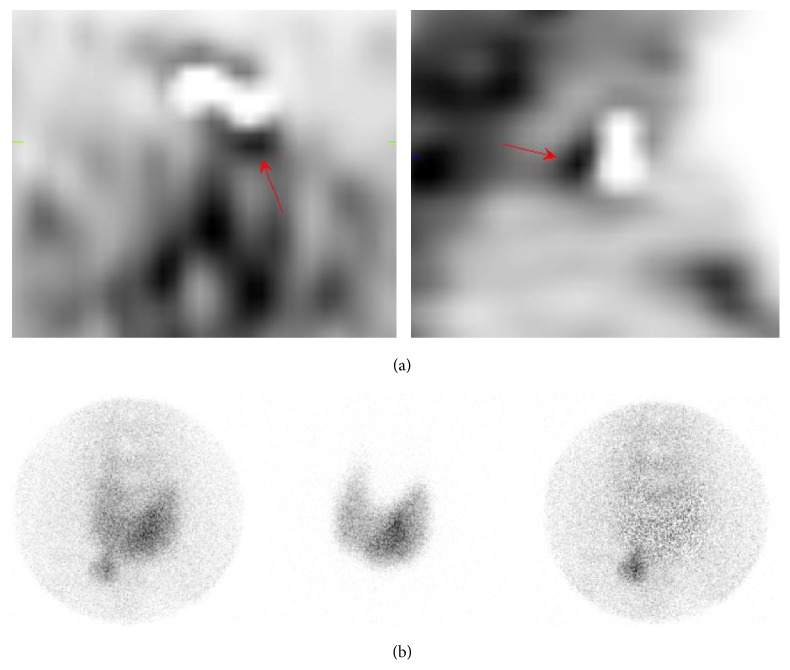
An example of a false-positive finding (case number eight in Table 3) on a ^99m^Tc-sestamibi/^123^I SPECT/CT due to an edge artifact (arrows). Oversubtraction is intentional to demonstrate the effect. An additional pinhole image showed no residual activity and was correctly classified as true negative. (a) Subtraction SPECT/CT images (*left*: transaxial;* right*: sagittal). (b) Anterior pinhole images (*left*: ^99m^Tc-sestamibi image;* middle*: ^123^I image;* right*: subtraction image).

Second patient (case number 10a/10b in Table 3) had 3 positive findings with both SPECT/CT and pinhole imaging. The upper right gland (small hyperplastic gland) was removed in surgery and classified as true-positive. Lower glands were visualized in surgery but not removed. Postoperative Ca-ion and PTH levels were normal. These two false-positive findings were classified as subtraction artifacts. Patient cases with false-positive findings are presented in Table 3 and in Figures 2 and 3.

## 4. Discussion

This retrospective study compared ^99m^Tc-sestamibi/^123^I protocols using SPECT/CT alone or combined with an additional anterior pinhole image. The results indicate high sensitivity and specificity for both protocols. The use of the additional anterior pinhole image with SPECT/CT was able to improve both sensitivity and specificity, although the difference was not significant. The sensitivity and specificity of ^99m^Tc-sestamibi/^123^I protocols using SPECT/CT alone or with pinhole imaging were equivalent in two recent studies [7, 18]. However, no comparison to a combination of these protocols was made in either of the studies.

Interesting results were published recently by Bhatt and colleagues showing that a ^99m^Tc-sestamibi/^123^I pinhole protocol detected more adenomas than the ^99m^Tc-sestamibi/^123^I SPECT/CT protocol and missed fewer adenomas than either the ^99m^Tc-sestamibi/^123^I SPECT-CT protocol or the combined pinhole and SPECT-CT protocol [13]. As Bhatt and colleagues suggested, one explanation for this result might be the rapid washout-phenomena since the SPECT/CT was started 45 minutes after sestamibi injection in comparison to five minutes in this study. Another explanation could be related to the acquisition parameters of the SPECT, since imaging was performed with quite a large angular sampling (5.6°), resulting in a very short acquisition time. SPECT reconstruction parameters could also have had some effect, but this possibility cannot be evaluated because the parameters were not published. We have seen in our clinical work that the reader's level of experience has a great effect on the sensitivity and specificity of ^99m^Tc-sestamibi/^123^I SPECT/CT. This could possibly have had some effect in the study by Bhatt and colleagues, since their study was conducted soon after the installation of SPECT/CT and the reader may not have had sufficient time to train with the new protocol.

### 4.1. The Importance of Pinhole Imaging

The EANM parathyroid guidelines stated in 2009 that* “although SPECT and SPECT/CT imaging are becoming very helpful, they cannot replace the standard planar and ‘pinhole' protocols that are still essential for optimal resolution in the thyroid bed region and for a correct diagnosis”* [10]. This is still true, since the spatial resolution of the pinhole collimator cannot be reached with any parallel-hole collimator. The highest achievable resolution is important especially for detecting the thyroid nodules, especially the sestamibi-hot/iodine-cold phenotype which may indicate thyroid malignancy [19, 20]. This phenotype also results in false-positive residual activity in subtraction images, which can be easily interpreted as parathyroid adenoma.

The importance of pinhole imaging was also highlighted in this study, as both sensitivity and specificity were higher with the combined pinhole protocol although the difference was not statistically significant. However, it should be noted that the number of false-positive findings decreased to half and four more adenomas were correctly localized with the pinhole imaging. Although not statistically significant, there was a change in the final outcome of this study for eight patients. The use of the additional pinhole imaging is fast; it does not increase the patient radiation dose or add to the total cost of the study. Thus we feel that the use of the additional pinhole imaging is justified to gain the highest possible sensitivity and specificity for each patient to avoid reoperation. It should also be noted that some enlarged glands are not obvious in anatomical CT image and may thus lead to false-negative outcome in SPECT/CT. Additional pinhole imaging may thus be helpful especially when a clinic is starting with ^99m^Tc-sestamibi/^123^I SPECT/CT.

### 4.2. The Edge Artifact

Even optimal processing of ^99m^Tc and ^123^I images does not give a flawless subtraction image but leaves some activity around the edges of thyroid lobes; this activity is called the “edge artifact” [7]. According to phantom measurements performed in our laboratory, this phenomenon could be reduced with careful implementation of acquisition and processing parameters, scatter/cross-contamination correction, and collimator selection [21]. However, further studies are needed in order to implement this solution in clinical practice.

### 4.3. False-Negative Findings

There were only three enlarged parathyroid glands that were not visualized even with the additional pinhole image. Two of these were small hyperplastic glands, one was a small adenoma (0.14 g) which was a part of a complicated patient case with a true-positive finding, true-negative finding, false-positive finding, and a false-negative finding (the patient had a thyroid cyst and a double adenoma).

### 4.4. False-Positive Findings

It has been suggested that SPECT/CT is superior to SPECT alone because the specificity of SPECT/CT is significantly greater than that of SPECT alone. The false-positive rate in this study with SPECT/CT was 6% which was in line with a Finnish study [6].

In addition to multimodality imaging, the use of ^123^I and subtraction images fused together gives a strong suggestion of the nature of the lesion in subtraction images. Also with additional pinhole imaging, we were able to avoid all false-positive findings due to iodine-cold nodules in the thyroid.

### 4.5. The Effect of Reconstruction

It has been shown in several publications that there are significant differences in image quality between commercially available algorithms and correction methods [22–24]. The effect of reconstruction parameters was not studied here. To further improve the quality of ^99m^Tc-sestamibi/^123^I SPECT/CT in parathyroid imaging, the final step is to optimize the protocol to include reconstruction and the correction methods for scatter, attenuation, depth-dependent resolution, and cross-contamination.

### 4.6. Limitations of the Study

Three different cameras were used in this study. All SPECT/CT acquisitions were performed with Siemens cameras (Symbia/Intevo) and, according to clinical quality control measurements, there were no differences in image quality. Pinhole acquisitions were performed with older Philips SKYLight with Siemens cameras. According to quality control measurements, there was no significant difference in image quality (resolution and sensitivity) between these cameras and thus no effect on results. The main bias in this study is probably the large variation in the time between scintigraphy and surgery.

## 5. Conclusion

This study indicates that ^99m^Tc-sestamibi/^123^I subtraction SPECT/CT is a highly sensitive and specific protocol for parathyroid scintigraphy. The use of additional anterior pinhole imaging increases both sensitivity and specificity of parathyroid scintigraphy, although this increase is not significant in this cohort. Given the low cost and short time required for additional imaging with no added radiation dose to the patient, we believe that the use of this additional pinhole imaging is justified to achieve the highest possible sensitivity and specificity for each patient to avoid reoperation.

## Figures and Tables

**Table 1:** Acquisition parameters for SPECT/CT and pinhole imaging.

Acquisition parameter	SPECT/CT	Pinhole
Detector configuration	180°	—
Orbit	Body-contour	—
Distance from the patient skin	—	10 cm
Collimators	LEHR	Pinhole, 5 mm opening
Matrix	128 × 128 matrix	256 × 256
Zoom	1	2,19
Pixel size	4.8 mm	
Acquisition mode	Step-and-Shoot	Static
Views over 360°	96 (3,75° per projection)	—
Time per projection or frame	33 s	10 min
Energy windows	140 keV 10%	140 keV 10%
	167 keV 10%	167 keV 10%
Voltage	130 kVp	—
Collimation	2 × 2.5 mm	—
Rotation time	0.8 s,	—
Pitch	1,5	—
Dose modulation	CARE Dose AEC + DOM	—
Reference exposure	80 mAs	—

**Table 2:** Sensitivity and specificity of ^99m^Tc –sestamibi/^123^I subtraction SPECT/CT and combined SPECT/CT with anterior planar pinhole imaging for all patients.

Patient group	Sensitivity/specificity	SPECT/CT	SPECT/CT with pinhole	*p*
All patients	Sensitivity	94%	97%	NS
	Specificity	98%	99%	NS

Primary hyperparathyreosis	Sensitivity	95%	98%	NS
	Specificity	98%	99%	NS

Secondary hyperparathyreosis	Sensitivity	89%	94%	NS
	Specificity	100%	100%	NS

NS indicates not statistically significant.

**Table 3:** False-negative and false-positive findings with ^99m^Tc –sestamibi/^123^I subtraction SPECT/CT alone and with an additional pinhole image. Case number with a letter (a and b) refers to the same patient.

Case number	Location	Clinical background	SPECT/CT	SPECT/CT with pinhole	Comment
1	LL	pHPT	FN	TP	Residual activity was faintly visible in SPECT/CT, but was classified as negative. With additional pinhole image correct classification was done.
2	UL	pHPT	FN	TP	
3a	LL	sHPT	FN	TP	
4a	LL	pHPT	FN	TP	

3b	LR	sHPT	FN	FN	No clear residual activity
5a	UR	pHPT	FN	FN	
6	UR	pHPT	FN	FN	

7	LL	pHPT	FP	TN	Very uneven iodine uptake
4b	UR	pHPT	FP	TN	Subtraction artefact (edge effect)
8	LL	pHPT	FP	TN	Subtraction artefact (edge effect)
9	LR	pHPT	FP	TN	An iodine-negative nodule

5b	LL	pHPT	FP	FP	A large thyroid cyst at left side
10a	LR	pHPT	FP	FP	Subtraction artefact
10b	LL	pHPT	FP	FP	Subtraction artefact

UL = upper left; LL = lower left; LR = lower right; UR = upper right.
